# Disability-related care trajectories in a pluralistic health system: a qualitative study in urban Sierra Leone

**DOI:** 10.1186/s12889-026-27861-9

**Published:** 2026-06-02

**Authors:** Hanna Luetke Lanfer, Elizabeth Anderson, Fatmata Bah, Phatima Mansaray, Franziska Feldkamp, Constanze Rossmann, Heleen Yoder, Anna Vines

**Affiliations:** 1 School of Public Health, Bielefeld University, Universitaetsstrasse 25, Bielefeld, 33615 Germany; 2 World Hope International, Enable the Children, Freetown, Sierra Leone; 3 Independent author, Freetown, Sierra Leone; 4 Department of Media and Communication, LMU Munich, Munich, Germany; 5 Nyandengoh!, Mattru Jong, Bonthe district, Sierra Leone

**Keywords:** Disability, Pluralistic healthcare, Traditional healthcare, Care trajectories, Sierra Leone, Qualitative Research

## Abstract

**Background:**

People living with disabilities (PLWD) often require long-term and repeated engagement with healthcare services. In pluralistic health systems, where biomedical, traditional, spiritual and NGO-based services coexist, care seeking is rarely linear and is shaped by multiple social, cultural and structural factors. Little is known about how disability-related care trajectories unfold over time and which factors shape access at different stages.

**Methods:**

This qualitative study is based on six focus group discussions (*N* = 42) with PLWD and caregivers of children living with disabilities, conducted in urban Freetown. Care trajectories were reconstructed from participants’ accounts and differentiated between early care seeking at symptom onset and long-term care. Qualitative content analysis was conducted, conceptualising access as shaped by people’s abilities to perceive care options, seek, reach, afford and engage with care.

**Results:**

Early care seeking was characterised by frequent movement between care domains, particularly biomedical, traditional and spiritual care, driven by uncertainty, trial-and-error processes and dissatisfaction with outcomes. Long-term care trajectories tended to focus on biomedical and NGO-based services, while traditional care was largely abandoned. Adult PLWD often disengaged from long-term care altogether. Access to biomedical care was shaped by substantial barriers, including physical inaccessibility, transport difficulties, informal payments, and experiences of discrimination. NGO services were perceived as more accessible, especially for long-term care yet were often unknown during early care seeking.

**Discussion:**

The findings illustrate how disability-related care seeking in a pluralistic health system is dynamic and shaped by evolving interpretations of disability, prior care experiences and structural constraints. A central tension emerged: biomedical care was perceived as more reliable for diagnosis, yet associated with the highest access barriers. NGO-based services, while experienced as more accessible, remained largely unknown during early care seeking.

**Conclusion:**

Improving access to disability-related care in pluralistic health systems requires attention to the full range of care domains that people navigate and to the structural and relational barriers shaping access over time. Strengthening accessible, respectful and coordinated care within public health systems, and increasing the visibility of NGO-based services at earlier stages of care seeking, are essential to support sustained engagement for PLWD and their families.

**Trial registration:**

N/A.

**Supplementary Information:**

The online version contains supplementary material available at 10.1186/s12889-026-27861-9.

## Introduction

An estimated one billion people globally live with some form of disability, a number that is expected to increase due to demographic ageing, chronic conditions and social determinants of health [1, 2]. The proportion of people living with disabilities (PLWD) is particularly high in middle- and low-income countries where exposure to hazardous working conditions, insecure housing, environmental risks, limited occupational protection and restricted access to preventive services all contribute to increased risks of injury, chronic illness and secondary health conditions [3–5]. Moreover, PLWDs tend to experience greater and long-term health needs than people without disabilities, including higher prevalence of secondary conditions and comorbidities, while at the same time facing heightened barriers to accessing healthcare [6, 7].

Disability encompasses a wide range of conditions, including congenital and early-onset impairments as well as disabilities acquired through illness, injury or conflict-related violence. Previous research in Sierra Leone has shown that these different forms of disability are framed and interpreted in distinct ways, including biomedical, social and spiritual explanations, which in turn shape how health needs are recognised and acted upon by individuals, families and communities [8–10]. Moreover, due to the chronicity of conditions, healthcare engagement for PLWDs and caregivers of children living with disabilities (CLWDs) often extends beyond discrete treatment episodes and persists over time.

Authors generally use ‘healthcare’ synonymously with modern, biomedical, evidence-based healthcare [11]. This is noteworthy as many countries, such as Sierra Leone [12, 13], employ pluralistic health systems. Pluralistic health systems have been defined as the coexistence of biomedical services with a range of other forms of care, including non-state, community-based, spiritual, traditional and complementary practices, as well as informal forms of self-medication [14–16]. Plural health systems hence reflect different epistemic traditions and forms of knowledge and influence how healthcare providers and patients interpret symptoms, seek relief and manage illnesses.

Sierra Leone is a low-income country in West Africa with a severely under-resourced public health system. Disability in Sierra Leone encompasses a broad range of conditions, including impairments related to the civil war (1991–2002), unsafe working and living conditions, and congenital or early-onset conditions that are often interpreted through spiritual and moral frameworks [8, 10]. Comprehensive, reliable prevalence data on disability and disability care remain scarce [17]. Research from Sierra Leone [13, 18–20] showed that patients (without disabilities or not specified) and caregivers consulted traditional and spiritual healers and biomedical health workers simultaneously or consecutively for different conditions or stages of diseases. These care-seeking behaviors were dynamically informed by cultural and socioeconomic factors, the availability and accessibility of providers, past experience with them, social and cultural norms, beliefs about the etiology of the disease, available financial resources and other factors. Thus, healthcare seeking in Sierra Leone is best understood as a fluid, cross-sectoral process in which families and individuals draw on a wide range of providers and services. As existing research has largely focused on general patient populations (especially maternal and children’s health [18, 19]), with limited attention to how PLWDs and caregivers of CLWDs navigate care options when health needs recur or persist, this study examines how PLWDs and caregivers of CLWDs navigate care trajectories over time within Sierra Leone’s pluralistic health system.

## Conceptual framework

### Care trajectories

In contrast to acute conditions, where healthcare engagement may be limited to a finite period, chronic illness and disability-related care often unfolds over longer periods and involves repeated interactions with different providers and services [2, 21]. To capture these dynamics, sociological and anthropological research has developed the concept of care trajectories, most prominently in the work of Strauss and colleagues [22, 23]. Care trajectories refer to the unfolding course of healthcare, welfare and social support over time, including the organisational work required to respond to health needs and the consequences this has for individuals and those involved in their care [22]. In doing so, the concept of care trajectories directs attention to how individuals and families navigate care across time and encompasses movements between different forms of care, phases of engagement and disengagement, and the ongoing reassessment of treatment options in light of perceived effectiveness, lived experience and changing circumstances [22, 24, 25]. Importantly, trajectories are often non-linear, contingent processes shaped by prior encounters with care providers, evolving interpretations of health conditions and the practical conditions under which care is sought and maintained.

In pluralistic health systems, care trajectories are further shaped by the coexistence of multiple therapeutic options that are evaluated and combined over time. Studies have shown that individuals and families draw on different providers as health needs change and as previous experiences inform expectations of care [26–28]. To capture how such patterns unfold in the context of disability in Sierra Leone’s plural health system, this study addresses the following research question:

- RQ1: What patterns of care trajectories can be identified among parents of CLWDs and PLWDs over time within the plural health system of urban Sierra Leone?

### Care trajectories through an access perspective

While the concept of care trajectories provides a conceptual vocabulary for describing how care unfolds over time, it does not in itself offer a structured analytical framework for analysing the conditions that shape access at different points along these trajectories and why specific care options are taken up, sustained or abandoned. Langwick’s [29] ethnographic work in Tanzania’s pluralistic healthcare system, for example, demonstrates how therapeutic choices were assessed from the perspective of those seeking care and based on factors such as observable changes (improvements or lack of change), the practical and relational dimensions of care encounters (e.g., distance and costs), and social recognition and acceptability of care providers. Understanding how people come to perceive healthcare needs, how they evaluate available options and how engagement is sustained or interrupted requires closer attention to the conditions under which care is accessed.

**Fig. 1:**
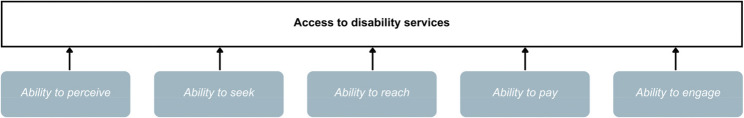
Ability dimensions of the access framework Note. Adapted by the authors based on Levesque et al. [30]

To address this, this study draws on the patient-centered healthcare access framework developed by Levesque, Harris and Russell [30], which was developed through the integration of earlier access models. Since its publication, it has been widely applied across different health conditions and settings, including in studies on chronic illness and disability, as documented in a recent scoping review [31]. The framework conceptualises access as a dynamic, relational process between health systems and careseekers, capturing both dimensions on the supply-side of healthcare services and the demand-side of careseekers. In this study, we focus on the latter. In their framework, the authors distinguish between five interrelated dimensions that influence access to care (see Fig. 1). *Ability to perceive* concerns the extent to which people can become aware that a source of care exists, can be reached and may be of potential relevance to their situation. It is influenced by the visibility of services in a community, the transparency of information about what is offered and the presence of outreach or informal information channels that make care options knowable. *Ability to seek* refers to the social and cultural alignment between services and users, including prevailing norms, beliefs and values about health, disability and legitimate forms of treatment, as well as perceptions of stigma, respect and trust in interactions with different kinds of providers. *Ability to reach* concerns the practical capacity to travel to healthcare facilities and attend appointments. Geographic proximity, transportation options, mobility determine this ability. Rural populations, people with disabilities, elderly individuals, and those with inflexible employment frequently face substantial barriers. *Ability to pay* reflects economic capacity to afford healthcare services. Current income, accumulated assets, insurance coverage, and understanding of financial protection mechanisms constitute this ability. Financial barriers at the point of service can prevent care utilization or force difficult trade-offs between healthcare and other basic needs. *Ability to engage* captures the capability to participate in treatment decisions and adhere to recommended care. Communication skills, language proficiency, self-management capacity, and social support for maintaining treatment all contribute.

Applied to the context of disability-related care in a plural health system, this framework allows for an analysis of the factors shaping access to care within those five dimensions, leading to our second research question:

- RQ2: Which factors shape access to healthcare along care trajectories for parents of CLWDs and PLWDs within the plural health system of urban Sierra Leone?

## Methods

### Study design

To examine care trajectories among caregivers of CLWDs and PLWDs in Sierra Leone, we conducted an exploratory qualitative study using focus group discussions (FGDs). FGDs were chosen to capture shared patterns of care-seeking and enable collective reflection within each group on experiences across different caregiving contexts. The study aimed to capture how participants described their pathways of care at different stages, from symptom onset and diagnosis to longer-term care seeking, as well as how these trajectories intersected with experienced barriers and enablers. The study was implemented through a collaboration between an academic research team, the non-governmental organization Enable the Children (ETC), which provides therapeutic and psychosocial services for CLWDs in Freetown, and two independent Sierra Leonean researchers (FB and PM) who were contracted for data collection. Importantly, the study was funded by an academic grant; the local researchers were affiliated with a university for the duration of the study and explicitly introduced themselves as independent university-based researchers and not as affiliated with the NGO. This approach was intended to reduce bias and power asymmetries related to service provision.

### Study setting

The study was conducted in urban Freetown, the capital of Sierra Leone and home to the country’s most concentrated landscape of formally available health services, including public hospitals, private clinics, NGO-based support and traditional and spiritual providers. This setting allows for an examination of how care trajectories and access barriers unfold in an environment where services nominally exist across multiple care domains.

### Recruitment

Participants were purposefully recruited into three analytically distinct groups. First, adult PLWDs who had no current or prior engagement with ETC or comparable formal support services were recruited through community-based approaches. One of the local researchers contacted potential participants directly in two suburban communities in Freetown, including individuals known within the community and individuals engaged in informal street-based activities such as petty trading or begging. Snowball sampling was used to identify additional participants within these networks. Second, caregivers of CLWDs who had been part of ETC’s programme for an extended period were recruited through ETC staff. Third, caregivers of CLWDs who had recently been enrolled in ETC’s programme, typically within the previous two to four weeks and who had not yet received substantive services, were also recruited with the support of ETC staff. Recruitment relied on existing programme contact lists and predefined criteria such as gender, length of involvement with ETC services and proximity to the location of the FGD. In assembling the groups, we aimed for a diversity of impairments; however, in the first group (adult PLWDs), only individuals who were able to represent their own perspectives orally were included, while those with intellectual disabilities or deafness were not part of this sample.

### Data collection

Data were collected through six FGDs conducted between April and May 2024 in four different suburbs of Freetown. Each group consisted of six to nine participants. FGDs took place in either hospital-based rooms used by ETC for service delivery and in community-based venues (community meeting places) (see Table 1 for an overview).

**Table 1:** Overview of focus group discussions

No	Date	Inclusion in ETC services	Group composition	Participants per group	Gender composition
FGD 1	18.04.24	Yes, for several years	Parents of a CLWD (mixed gender)	8	6 F 2 M
FGD 2	30.04.24	Yes, recently admitted	Parents of a CLWD (mixed gender)	9	7 F 2 M
FGD 3	03.05.24	No	PLWD (mixed gender)	6	2 F 4 M
FGD 4	04.05.24	Yes, recently admitted	Mothers of a CLWD	6	6 F
FGD 5	19.05.24	Yes, for several years	Fathers of a CLWD	6	6 M
FGD 6	19.05.24	No	PLWD (mixed gender)	7	4 F 3 M

All FGDs were conducted in Krio, the most widely spoken lingua franca in Sierra Leone. Each discussion was facilitated by two trained local researchers using a semi-structured discussion guide. The guide was jointly developed by the academic researchers, NGO staff and the local researchers, piloted in a test focus group, and subsequently refined. Topics included care-seeking pathways at different points in time, experiences with formal and informal providers and perceived barriers and facilitators in accessing care (see supplementary file). A short survey to capture participants’ sociodemographic characteristics (age, gender) and type of disability of oneself or the cared-for person was conducted with each participant individually either before or after the FGD. Information on impairments was recorded based on participants’ or caregivers’ descriptions, without assigning these descriptions to predefined diagnostic categories.

Prior to the discussions, all participants received an oral and written explanation of the study aims, data protection measures and voluntariness of participation. Participants were informed that participation in the study would not influence their eligibility for current or future services provided by ETC and that only anonymized data was shared with the NGO. Given varying literacy levels, the consent form was read aloud and explained in detail. Participants could ask questions and consult a trusted person if desired. Informed consent was documented via signature or thumbprint.

All discussions were audio-recorded with permission and lasted between 55 and 128 min. Transcription and translation into English were conducted simultaneously by the researchers who facilitated the discussions. This approach was chosen to preserve contextual nuance and reduce the loss of meaning. Where metaphors or culturally specific expressions lacked a direct English equivalent, explanatory notes were added to the transcripts. All identifying information was removed, pseudonyms assigned, and audio recordings deleted after transcription was completed.

### Data analysis

The analysis combined quantitative content description and qualitative analysis, supported by MAXQDA 2024 (VERBI GmbH). For the first research question, participants’ descriptions of their care trajectories were systematically reconstructed based on their accounts and differentiated between care seeking at symptom onset (first, second and third steps, depending on how many care-seeking steps were described by each participant) as well as subsequent long-term care (ongoing therapies or long-term routines after diagnosis). First, second and third steps refer to analytically distinct phases of care seeking (e.g., being admitted to a hospital for a week with no changes (first step), then seeking care with a traditional healer (second step)). In several cases, participants reported engaging with more than one care domain in parallel or in rapid succession. For example, several caregivers described interrupting an initial care-seeking attempt to seek care somewhere else before resuming the initial attempt (e.g., intending to go for biomedical care with a sick child, being stopped due to interventions by family or community members to seek help first from a spiritual or traditional healer, before resuming biomedical care later the same day). These cases were coded as simultaneous care seeking with more than one care domain assigned to the same step. All described steps were assigned to care domains derived from the data, including biomedical care (e.g., governmental and private hospitals and clinics), traditional care (e.g., traditional healers and herbalists), spiritual care (e.g., pastors, imams), NGO clinic/ services (e.g., ETC), self-medication/ treatment (e.g., buying drugs from a pharmacy), and no care seeking (coded when participants explicitly described not engaging with any form of care at a given stage (e.g., waiting for symptoms to disappear at symptom onset or disengagement from care during long-term care)). For each participant, their sequence of care-seeking steps was documented in an Excel sheet and aggregated across cases.

For the second research question, all focus group data were analysed using qualitative content analysis following Kuckartz and Rädiker [32]. An initial coding frame was developed deductively based on the five dimensions of Levesque et al.’s access framework [30] – ability to perceive, seek, reach, pay for, and engage with healthcare – which served as the main analytical categories. Within each of these categories, subcodes were developed inductively from the material, capturing patterns and themes that emerged from participants’ accounts.

Coding was conducted by HLL and a student research assistant (FF). In the initial phase, both coded two different transcripts to identify new subcodes. After refining and finalising the coding structure, the full dataset was coded independently by both coders. Coding results were then reviewed and discrepancies were resolved through discussion. Regular discussions with the other team members served to reflect on emerging interpretations, resolve ambiguities and ensure contextual plausibility of analytic decisions.

### Positionality

The research team is an all-female team and combines NGO-based and international academic perspectives with locally grounded research expertise. The German and NGO-affiliated team members – all with long-standing professional engagement in Sierra Leone and experience in disability-related work – were responsible for study design and analysis. The two Sierra Leonean researchers (FB and PM), both women with qualitative research experience in contract-based research roles, played a central role in the development of the discussion guide, participant recruitment, data collection, and contextual interpretation. None of the authors identify as living with a disability or has a CLWD. The team engaged in ongoing reflexive discussions about how institutional affiliations, gender and other social positions within the team influenced participant interactions, team collaboration, analysis and representation of findings.

## Results

In this section, we first describe the sample demographics (Table 2), followed by the results for each of the two research questions. To facilitate understanding, quotes from the discussions have been translated into English. Krio is a distinct language with its own grammar and structure, though it has roots in English. In translating participant quotes, we have aimed to preserve their original meaning and tone while making minor adjustments for clarity where necessary.

**Table 2:** Demographics of study participants (*N* = 42)

Characteristics	*n*	%
Gender of participant		
Female (F)	25	60
Male (M)	17	40
Participant category		
Caregiver of CLWD	29	69
PLWD	13	31
Disability of the participant’s child or the participant*		
Amputation/ limb/ mobility impairments	23	55
Speech impairment	8	19
Polio	7	17
Developmental delay / learning difficulties (caregiver-reported)	7	17
Cerebral palsy	5	12
Sensory impairments (deaf/hearing impaired, blindness)	3	7
Muscular dystrophy	1	2

* Note. Multiple impairments could be reported per participant

### Sample demographics

A total of 42 participants were included in our study (see Table 2 for participant demographics). The sample consisted predominantly of women (60%) and caregivers of CLWDs (69%). The age of respondents ranged from 18 years to 46 years and older, the majority were between 26 and 35 years (36%). The sample included caregivers of CLWDs and PLWDs who presented various disabilities: Limb and mobility impairments were the most prevalent, affecting 23 individuals (55%), followed by speech impairments (19%) (only among CLWDs) as well as polio (17%) and developmental delays (17%).

### Patterns of care trajectories over time (RQ1)

**Fig. 2:**
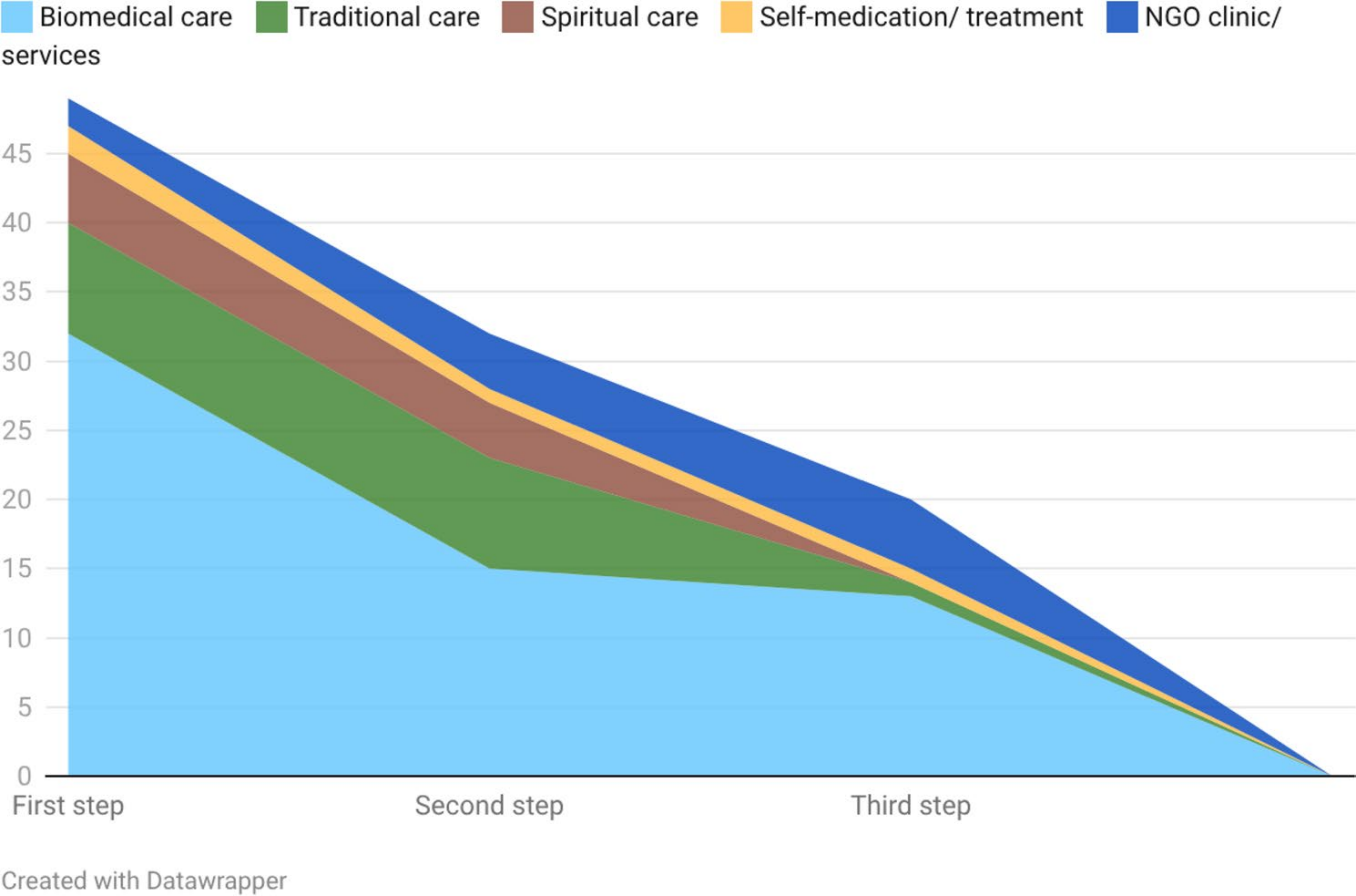
Patterns of care seeking at symptom onset (a)

Patterns of care trajectories were divided into (a) symptom onset or first recognition of symptoms and (b) long-term care. At symptom onset, the majority of participants described multiple, sometimes overlapping care-seeking steps: in total, 51 first steps were coded across 42 participants, indicating that nine individuals engaged with more than one type of care simultaneously; 32 participants reported a second step and 20 described a third step in their early care-seeking process (see Fig. 2 and corresponding table in the supplementary file).

At symptom onset, the majority of participants reported initially seeking biomedical care at a hospital or a health centre as the first step (n = 32). Here, different forms of symptom recognition leading to care seeking were described. In some cases, onset was experienced as an acute event associated with urgency, for example convulsions or sudden illness, while in other cases concerns emerged more gradually, such as when children did not start to sit, walk or speak as expected. Concurrently or shortly thereafter, several participants also reportedly engaged with spiritual (n = 5) or traditional (n = 8) forms of care. During this phase, care-seeking practices were frequently characterised by parallel engagement with, or rapid movement between, biomedical and traditional or spiritual forms of care. Seeking biomedical care from more than one facility was also commonly reported as a second (n = 15) or third (n = 13) point of contact, either because the first facility had referred the patient or as participants were not satisfied with the treatment or diagnostics of one facility.

“We went to the hospital [in Freetown], nothing was done and they decided to send us to [hospital in the northern province]. They tested him but they said that they did not see any illness in him. They sent us to [other hospital in the northern province], we stayed there for a month and still they did not able to see or detect any sickness on him. So we came back and started the traditional approach.” (P4, FGD2)

For a subset of participants, the first steps to seek care took place within traditional care settings (*n* = 8). Over time, however, a distancing from traditional care was frequently observed: participants reported (re)turning to biomedical services after using traditional or spiritual care, particularly when symptoms worsened or when these treatments were perceived as ineffective or burdensome. Care seeking from NGO clinics, self-medication or not seeking treatment were rare during the phases of symptom onset or observed symptoms. No notifiable differences were observed between the FGDs.

Moreover, many participants described the early stages of their care trajectory as marked by uncertainty, frustration and difficulties in finding appropriate care as the quote from a mother shows:

“It was at the age of nine months that she was attacked by convulsion. So, I was going through that stress. I took her to the hospital and they [hospital staff] said nothing is wrong with her. So, I had to try the traditional way to cure the sickness. You know that the traditional treatment has a lot of things to be done and people said a lot I should do, but I don’t know what was actually right.” (P8, FGD2)

**Fig. 3:**
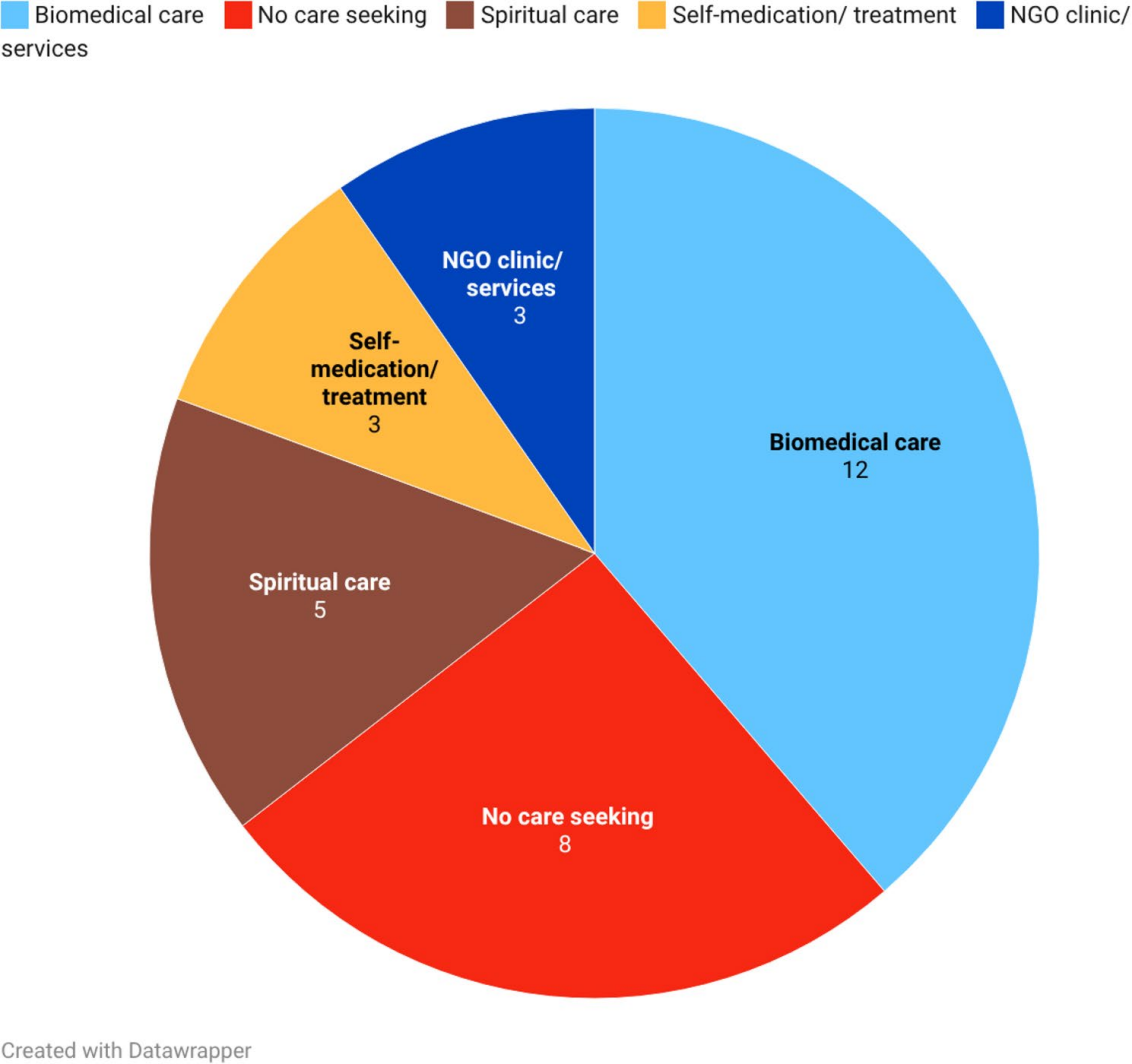
Patterns of long-term care seeking (b)

Regarding long-term care seeking (see Fig. 3), it became evident that care trajectories had changed for most participants since the early steps of care seeking with a shift from attempts to “cure” the condition towards forms of care aimed at managing or living with the disability. The utilisation of biomedical care and NGO services dominated long-term care for services such as physiotherapy, follow-up consultations or other measures aimed at maintaining mobility, functioning or preventing deterioration. A growing number of participants described no longer engaging in care-seeking (*n* = 8) or doing self-medication (*n* = 3). Traditional care was not reported as part of participants’ long-term care trajectories whereas spiritual care was sought in some cases as part of long-term trajectories (*n* = 5).

Comparison across the FGDs indicated distinctions in long-term care. Parents of CLWDs, particularly those involved in NGO services for several years (FGDs 1 and 5) reported more frequent and sustained engagement with care services. These caregivers described that they knew where to seek assistance and experienced greater continuity in care provision. In contrast, PLWDs more commonly reported irregular patterns of care use over time. Their trajectories were characterised by periods without active care-seeking and limited to situations in which additional health problems or comorbidities occurred, withdrawal from formal healthcare structures and persistent frustration resulting from prior care experiences.

### Experiences of access to healthcare (RQ2)

Participants highlighted a wide range of factors that shaped how, when and why healthcare was accessed or discontinued over the course of care trajectories. These factors are described here in line with the five interrelated dimensions outlined in Levesque et al.’s access framework [30]: the ability to perceive, seek, reach, pay for and engage with healthcare. An overview of the coding scheme can be found in Table 3.

**Table 3:** Overview of the coding structure

Ability domains	Subcodes	Themes
Ability to perceive: Awareness of care options and access to information	Visibility of care options	General entry points known Entry points into specialised forms of care hardly known
	Information sources and channels	Family and peer networks shaped who and in which order providers were consulted
	Informational exclusion	Disabilities can limit access to information (mobility, hearing or speech impairments Desire for outreach targeted at PLWD
Ability to seek: Weighing meaning of disabilities, acceptability and social consequences of care options	Explanatory models of disability	Different explanations for causes of disability, including congenital, wrong treatment, following a disease, witchcraft, spiritual attack
	Perceived acceptability of providers	Respectful communication & encouragement fosters acceptability Dismissive and discriminatory behaviour discourages acceptability
	Anticipated consequences of care-seeking	Stigma from other patients (biomedical) Family conflicts arising from disability attribution (traditional)
Ability to reach: Navigating physical, organisational and infrastructural access	Availability of appropriate services	Often unavailable or difficult to reach (biomedical) Desire for healthcare workers with disability experience
	Transport and logistics	Challenges in getting public transport to biomedical facilities Proximity of healers increase accessibility (spiritual and traditional)
	Physical accessibility	Poorly equipped for the needs of people with physical impairments/ not barrier free (biomedical) Gatekeeping and obstruction at hospital entrances (biomedical)
	Waiting time for treatment	Long waiting times until being attended to (biomedical)
Ability to pay: Affording care amid formal and informal costs	Informal payments and hidden costs	Demands for a bribe/ token by different hospital staff (biomedical)
	Costs for medicine and consultation	Free services and support for transportation (some NGO services) Costs despite free healthcare for medicine and consultation fees (biomedical) Overcharging and intransparent costs (traditional)
Ability to engage: Staying involved in diagnosis, treatment and long-term care	Perceived accuracy of diagnosis and targeted care	Tests and specialised assessment (biomedical and NGO services) Rapid and unsubstantiated diagnoses (traditional)
	Perceived effectiveness of treatment	Perceived improvement (biomedical, traditional and spiritual) Skepticism about traditional treatments Deterioration or no improvement (biomedical, traditional and spiritual)
	Participation in treatment	Involvement in treatment routines, especially in long-term care (NGO services) Being treated as partners (NGO services)
	Competing priorities	Daily chores as an obstacle to long-term care Costs as barriers to care

### Ability to perceive: awareness of care options and access to information

Participants’ descriptions of how they became aware of available care options were subsumed into three subcodes: *visibility of care options*,* information sources and channels and informational exclusion.*

*Visibility of care options* referred to the recognisability and presence of care options in participants’ surroundings. Especially at symptom onset, PLWDs and caregivers of CLWDs reported that they did not initially know where to seek help. While most participants were familiar with hospitals and traditional healers as general providers, entry points into specialised forms of disability-related care were not known.

*Information sources and channels* were predominantly informal and interpersonal: family members, neighbours and peers were often the most influential sources when initial care options were considered. These informal pathways shaped how different types of care became known or were evaluated and sometimes in which order providers were consulted:

“For me, they [people in her social network] told me to use the native or traditional approach but my husband said no because he doesn’t believe in those things. So he said that I should take the child to the hospital where I gave birth to the child.” (P6, FGD1)

While some participants benefited from the information they received from their interpersonal networks, none described structured health education or public outreach.

*Informational exclusion* was reported by several PLWDs and caregivers. Participants with mobility impairments reported that physical access to hospitals, public places or community gatherings was often limited and thus reduced their opportunities to obtain relevant knowledge. Others pointed to challenges related to digital literacy, the absence of smartphones or internet access and expressed a wish for better communication targeted at PLWDs:

“It will be good to provide a special way for persons with disability where they can be informed about how they can have access to their treatment. Like for instance if there is a megaphone to announce to them in the community where they can go for their treatment.” (P8, FGD1)

### Ability to seek: weighing meaning of disabilities, acceptability and social consequences of care options

Participants’ descriptions of how they decided whether and where to seek care were structured around three subcodes: *explanatory models of disability*,* perceived acceptability of providers*,* and anticipated social consequences.*

*Explanatory models of disability.* Participants drew on different interpretations to make sense of disability, often combining biomedical, spiritual and traditional explanations. These shaped the perceived choice of care options and influenced when and how providers were consulted. Particularly among parents of CLWDs with early onset disabilities, uncertainty about the origin of the condition led to sometimes repeated shifts in interpretation of the condition over time and some expressed frustration at the lack of clarity or formal diagnosis.

“He [traditional herbalist] said that it was a devil that gave me the pregnancy. So I told my mother that we should leave because I know that it was not a devil that gave me the pregnancy.” (P6, FGD2)

Participants in FGDs with NGO contact described the causes of disability in biomedical terms, e.g., congenital causes or following an illness. However, biomedical treatment or rather malpractice and treatment-related harm were also described as a cause of disability:

“I was not born disabled. According to my parents, I was close to six years and I went to sleep one evening. I woke up and I was feeling fever. They [parents] took me to the hospital and the nurse gave me an injection and after that injection I was not able to stand again. Since then I have not been able to walk again.” (P5, FGD6)

Across FGDs, participants’ explanatory models of disability appeared to evolve over time. Several participants explained that they did not simply adopt what providers told them about the cause of disability and respective treatments or cure but also evaluated such statements based on personal beliefs, social context and observed changes in the condition over time.

*Perceived acceptability of providers.* Experiences of provider behaviour shaped whether participants perceived a type of care as acceptable and whether they were willing to continue using it. Several caregivers highlighted how important respectful communication and encouragement had been to them and supported their care seeking.

“At first, because of the condition of my child, I was very ashamed to bring her outside. …When I came to the hospital, they comforted me. I thought my child is not going to walk, it was my first born and I had never seen such thing like this in my life, but all the staffs encouraged me so I took courage.” (P2, FGD4)

However, these positive experiences stood in contrast to widespread accounts of dismissive and discriminatory behaviour by both biomedical (this was the most frequently coded theme in our analysis; all but three participants described such experiences) and traditional providers. In biomedical facilities, participants repeatedly reported being treated as unimportant, ignored or actively discouraged. Financial pressure often intersected with these experiences, with caregivers sensing that their social status or ability to pay influenced how they were treated.

“It seems like we all have the same story of discrimination. At the look of things you do not have the required finance they [nurses] wanted from you and out of this desperation, they don’t seem to be pleasant.” (P5, FGD5)

“They [hospital staff] don’t even talk to me and my child nicely and I don’t feel good about that as a mother.” (P6, FGD2)

Similar patterns were reported in traditional care. Participants described how herbalists made demeaning statements, such as attributing disability to demonic causes or suggesting the child should be excluded from their family’s life or even abandoned (e.g. ceremony/ritual that involves leaving the child in the forest) based on the beliefs that it is a spiritual being (e.g. a ‘snake’ or ‘devil’) that should be returned to the spirit world.

“Sometimes when we go to the native doctors, they discourage you, like for instance, they will tell you that your child is a devil and you should not sleep with a devil at your side. Or you should return the child” (P1, FGD2)

While biomedical providers in our study were criticised for coldness and lack of attention, traditional providers were more often described as reinforcing harmful narratives about the cause of disabilities. In both cases, these experiences could lead to withdrawal from care.

*Anticipated consequences of care-seeking.* In addition to direct provider behaviour, participants described how anticipated reactions from others influenced their ability and willingness to seek care. Stigma from other patients in hospitals was reported repeatedly and described as emotionally painful and isolating:

“Some of them [other patients] behave to us as if we are not fit to be among them or as if we created the problem for ourselves. Most of the other parents discriminate our children. So all these things sometimes make me feel bad to the point that I don’t want to go to the hospital.” (P2, FGD1)

In traditional settings, anticipated social consequences were often linked to intra-household dynamics. Several participants recounted that spiritual or traditional explanations of disability led to blame being assigned to mothers or fathers – in some cases undermining family relationships:

“When you go to the traditional side, he [herbalist] will start by telling you, you are the one with the problem or your wife is the cause of the problem of the child. He creates enmity between your wife.” (P2, FGD5)

### Ability to reach: navigating physical, organisational and infrastructural access

Participants’ descriptions of access barriers were structured around four subcodes: *availability of appropriate services*,* transport and logistics*,* physical accessibility of facilities* and *long waiting times.*

*Availability of appropriate services.* Across FGDs, described the need for specialised care for disabilities, e.g. hospitals only for PLWDs. However, they also reported that relevant, specialised services were often either unavailable or only provided in distant hospitals which required the patient to travel there. Even when facilities were available, they were often experienced as functionally limited. Moreover, it was mentioned that health workers with a disability were almost non-existent and participants viewed providers ‘like oneself’ as an improvement to disability services:

“We are appealing to the government to employ disabled nurses and doctors, because if I go to the hospital and see my disabled colleagues, I will feel free.” (P3, FGD6)

*Transport and logistics* were repeatedly mentioned as a major challenge, particularly in relation to getting public transport to biomedical facilities. Caregivers and PLWDs reported that drivers often refused to take them or charged higher prices.

“When you stand waiting for transport with a disability person, they will push you aside, saying you are not a better person and allow the normal people to enter the vehicle.” (P3, FGD3)

For some participants, the proximity of traditional healers offered a more accessible alternative. In several accounts, traditional care was described to be located within walking distance or provided in people’s home, making it more attractive in situations where transport to hospitals was unaffordable or logistically complex:

“There was a man in our neighbourhood who was helping me with native medication and massages. We did this for almost three years and it [hunch back] became more straight and she started to sit down even though the neck was not straight.” (P6, FGD2)

*Physical accessibility* to biomedical healthcare facilities was another frequent concern. Participants described hospitals as poorly equipped for the needs of people with physical impairments. This affected both adults living with disabilities and caregivers of children with mobility issues:

“They [people with mobility impairments] cannot stand on their own or take care of themselves. So, I put the child on my back if I want to go and use the restroom and sometimes I will take someone along with me to assist me getting around in the hospital.” (P4, FGD1)

In several cases, participants described gatekeeping and obstruction at hospital entrances, where guards demanded them to use other entrances which added another layer of access difficulty especially for people with mobility impairments:

“They will tell us to use the back gate and there, they will ask you to give them money before they open the gate for you.” (P1, FGD1)

Finally, *waiting time for treatment* emerged as a recurring theme. Several participants reported that they were made to wait for hours in uncomfortable positions, regardless of the severity of their child’s or their own condition. Some described being deprioritised or told that their case was less urgent than others:

“You go to the hospital and you have to wait hours and hours before you are attended to. The doctors, they tell you to wait over there like, ‘your problem is different and we are treating other people first.’” (P2, FGD3)

Caregivers of CLWDs explained that due to the often experienced long waiting times, they had to neglect household chores or income generating activities, which further caused an organisational barrier.

### Ability to pay: affording care amid formal and informal costs

Participants highlighted a range of financial factors influencing access to care, captured in two subcodes: *informal payments and hidden costs and costs for medicines and consultations*.

Participants across all FGDs described financial factors as a central barrier to accessing and continuing care. While some reported receiving free services and even support for transportation costs, especially through NGO-supported facilities, most participants referred to *informal payments and hidden costs* encountered in biomedical settings. These included bribes at hospital gates to security staff and to nursing staff inside the hospitals.

“Wherever you go, when you want to get treatment, they will ask you to give money and you must give that 10,000 or 20,000 Leones before they could even give you attention.” (P2, FGD6)

“If you don’t give them [nurses] a tip, they will not attend to you. They forget that they are there to save life – not to do business!” (P4, FGD6)

Participants also described *costs for medicine and consultation*, despite the nominal existence of free healthcare. Several reported being unable to afford prescribed tests or treatment, which led to delays, incomplete care or partial treatment based on what was financially possible:

“The tests that my child is supposed to do and get for free, if I don’t have the money, they will not do the test for my child. So, most times, I prefer them to treat my child for what I can afford.” (P1, FGD1)

Costs were also a concern in traditional care settings and these were described as intransparent and overcharged in traditional care by several participants:

“When I took that adventure to seek medical for my child from a traditional healer, she demanded plenty things, fowl, rice, money oh! It was too much!” (P3, FGD5)

Nonetheless, in other cases, traditional care was described as financially accessible, particularly in comparison to biomedical care.

Across all groups, the cost of care was experienced not just in terms of affordability, but also unpredictability. Participants rarely knew in advance how much a treatment would cost, and reported feeling pressured to pay without fully understanding what services they were receiving. The exception were NGO-connected participants who often described receiving care without payment.

### Ability to engage: staying involved in diagnosis, treatment and long-term care

Participants’ accounts of how they interacted with providers and stayed involved in treatment processes were structured around four subcodes: *perceived accuracy of diagnosis and targeted care*,* perceived effectiveness of treatment*,* participation in treatment and competing priorities.*

*Perceived accuracy of diagnosis and targeted care.* Participants described differences in how accurately they felt their condition or that of their child was diagnosed and whether care was perceived as appropriate and targeted. In several accounts, particularly among caregivers with long-term NGO support, participants felt actively included in diagnostic processes and described trust in the care suggested.

“We have two children with disabilities and we were convinced to go the traditional way, but it was the worst decision we took. … At the hospital, we were both interviewed, they talked to us for a long time. My wife narrated her story about her brother who were disabled from childhood to adulthood until he died. It was there that they made findings that the sickness could be coming from my wife’s family.” (P3, FGD5)

In contrast, several participants described traditional providers as offering rapid but unsubstantiated interpretations of symptoms, presenting diagnoses with confidence but without any form of examination or explanation which made them doubt the treatment:

“The hospital has different specialised doctors that can treat different parts of my body. But for traditional healers they don’t do a test to know what is exactly wrong with you. They will just give you the treatment blindly.” (P6, FGD6)

Participants also spoke about the *perceived effectiveness of treatment*, which influenced whether care was sustained. Positive observed outcomes were reported from both biomedical and traditional treatment, though participants were particularly skeptical about traditional treatments and the way it had an impact:

“Well, the medication that he was preparing looked funny because he was just using leaves to write from the Quran and then rub the leave on the body of the child. … I was surprised it got a little better.” (P8, FGD2)

Negative outcomes were also described in both care settings. Several participants referred to no improvement or deterioration after receiving treatment, which led them to stop or switch providers:

“My mother decided to take me to the hospital every morning, I would be given injections and injections, but we couldn’t see any improvement and my mother said she is going to stop.” (P4, FGD3)

“At the traditional, it was the worst. The herbalist used herbs to rub all over the child and the sickness became worse.” (P3, FGD5)

*Participation in treatment.* Some participants described being actively involved in treatment routines, received clear instructions and were treated with respect. This applied especially to physiotherapy and home-based care, where continued engagement depended on mutual communication, trust and the caregiver’s own capacity. In NGO-supported settings, caregivers more often reported that they were included as partners in the treatment process and received follow-up or advice over time. Where this was lacking, care remained fragmented and participants navigated support systems with little guidance or continuity.

*Competing priorities* were described as a key influence, mostly by caregivers. Caring for a CLWD often required time and energy that conflicted with other responsibilities such as income generation or household duties. Even when services were available and free of charge, participants reported being unable to follow through with home-based care due to lack of time, support or physical energy.

“Well, most of us the parents, we don’t do the exercise that they tell us to do. With a disabled child that can’t do anything on their own, it’s not easy. There is always something that needs to be done and sometimes you just cannot manage everything in a day.” (P3, FGD1)

Moreover, the high costs (see ability to pay) were also repeatedly mentioned as a barrier to sustained long-term care.

## Discussion

This focus group study examined how people living with disabilities (PLWDs) and caregivers of children living with disabilities (CLWDs) (*N* = 42) in Freetown, Sierra Leone navigate the pluralistic health system over time (RQ1) and what shapes their access to care (RQ2), using the healthcare access framework proposed by Levesque et al. [30] for analysis.

Findings related to care trajectories (RQ1) highlight that care seeking often involved shifts between different care domains, particularly at the onset of symptoms when most participants described to have engaged with at least two different care options. These patterns highlight the fragmented and cross-sectoral nature of Sierra Leone’s pluralistic health system, in which different providers co-exist with few structured referral systems between them [12, 13]. Participants described changing providers based on available options, urgency of symptoms, advice from others, financial constraints or dissatisfaction with outcomes. This confirms findings from other pluralistic settings, where care seeking is shaped by uncertainty about care options, availability, past experiences and social influences [33–35].

A notable shift was observed when participants described their long-term care arrangements. While biomedical care dominated early responses to symptoms, NGO-based services became more prominent over time. In these cases, NGO services appeared to stabilise care trajectories and offer a clearer structure for referrals and continued support, hence functioning as a bridge between different sectors and services. At the same time, nine participants, all from the PLWD focus groups, reported disengagement from care structures altogether. In our study, these accounts were often shaped by past experiences of frustration, limited improvement by care providers and individual coping mechanisms, but may also reflect structural gaps in rehabilitative and long-term services that offer fewer options for adults compared to children living with disabilities [34, 36, 37].

Across the five access domains (RQ2), a notable paradox emerged: biomedical care was often described as the most accurate, specialised and desirable form of treatment, particularly due to its diagnostic capacity and perceived effectiveness. Participants referred to tests, professional assessments and targeted interventions as key strengths of biomedical services, especially in comparison to traditional providers, whose explanations were seen as vague or arbitrary. Yet, despite this epistemic legitimacy, biomedical care was simultaneously associated with the highest barriers to access. These barriers spanned all five ability dimensions according to our used framework [30]: long distances to reach facilities and difficulties in obtaining public transport, physical inaccessibility of the facilities, extended waiting times, informal gatekeeping practices and unpredictable costs. Participants often had to make ad-hoc decisions about whether and how to seek care, without knowing in advance what they would have to pay, when they would be treated or whether services would even be available. These access constraints were compounded by experiences of discrimination, verbal abuse and social exclusion in public biomedical facilities as participants reported feeling ignored, belittled or actively discouraged by staff, especially when they were unable to pay or were perceived as socially marginal. A combination of obstacles in biomedical healthcare access is also well documented in the literature (e.g [35, 38–41].

Our findings also resonate with other research in how access to care was shaped by intersecting social positions, including gender, disability status, poverty and family context [39, 42]. In particular, several mothers of CLWDS described being mainly responsible for household duties, caregiving for multiple family members and seeking healthcare for the CLWD. These responsibilities were further exacerbated by often limited financial means and structural dependencies to their husbands or other family members which further constrained their ability to seek care autonomously [8, 43, 44].

In sum, this study illustrates how healthcare seeking is an active, continuous process of meaning-making and (re)assessment. Participants rarely followed the advice or diagnoses of care providers unquestioned. Instead, they evaluated, negotiated or rejected different diagnoses and treatments based on their own observations, prior outcomes, beliefs, affordability, availability and social resonance. These findings highlight caregivers’ and patients’ reasoning, agency and their abilities to perceive, seek, reach, pay for and engage with care in a pluralistic system [28, 45]. In this sense, participants’ accounts can be viewed as health literacy, namely the ability to interpret information, appraise options and make context-sensitive decisions in a system in which no single care model is dominant [46].

**Fig. 4:**
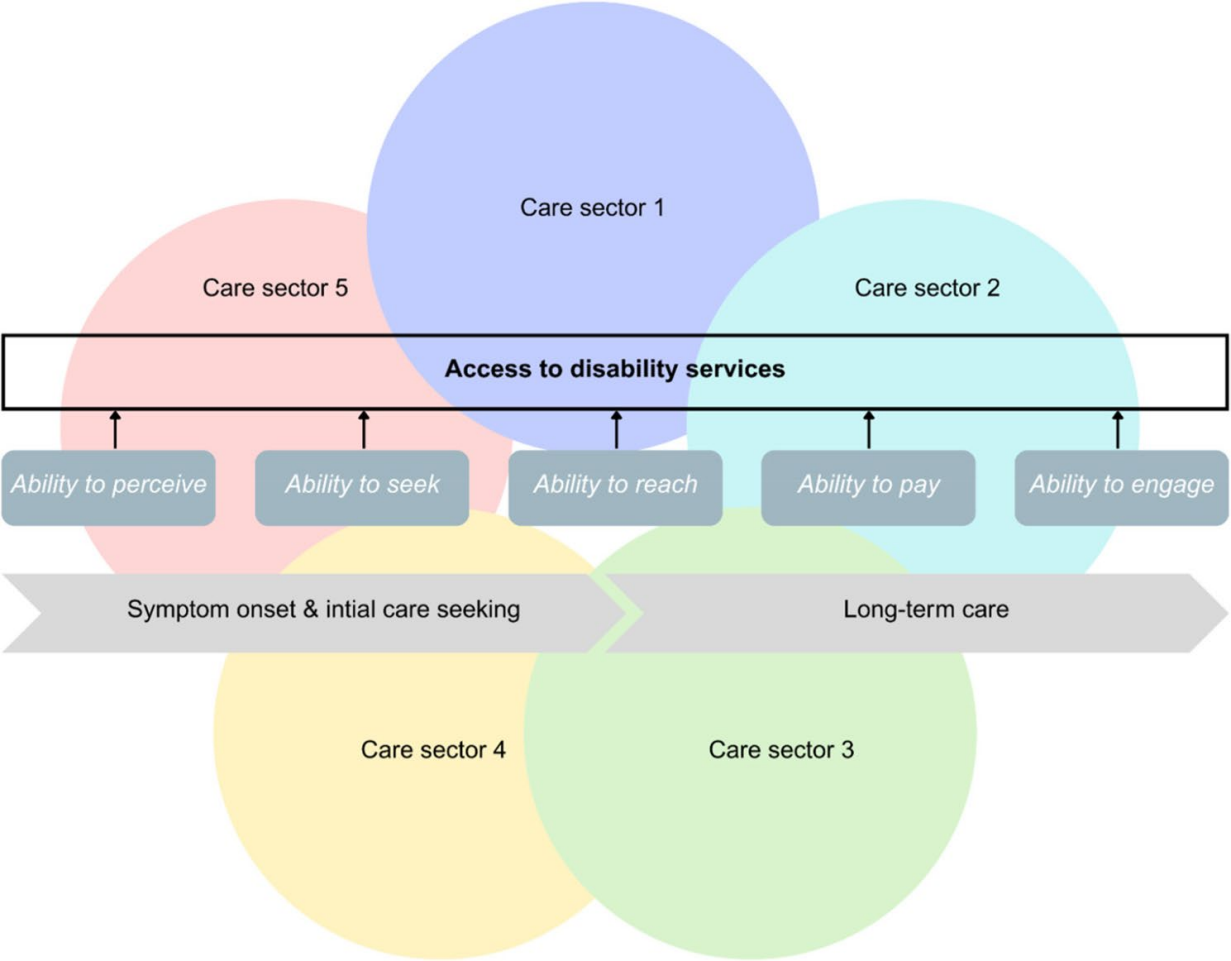
Schematic extension of the access framework in a pluralistic health system

This study contributes to the further development of the healthcare access framework by Levesque et al. [30]. First, our analysis extends the framework to explicitly pluralistic health systems by showing that access cannot be conceptualised in relation to a single sector but must be understood across coexisting biomedical, traditional, spiritual and NGO-based care domains that are navigated in relation to one another. The original framework was predominantly applied in contexts of formal, biomedical healthcare provision and not to address such cross-sectoral navigation [31]. Second, while this access framework is typically used to investigate or explain access to specific services or at particular points in time, our combination with a care trajectory perspective demonstrates that the five abilities evolve over time and in response to prior care experiences, shifting interpretations of illness and changing life circumstances. Figure 4 illustrates this conceptual extension. It visualises access as a dynamic process unfolding over time, shaped by different exemplary coexisting care domains that intersect with the five access abilities. The schematic structure allows for applications beyond the specific case of Sierra Leone, including other pluralistic health systems.

Taken together, the findings underline the importance of approaching pluralistic health systems as a structuring condition that shapes how care is accessed, interpreted and sustained [28]. In international discourses, ‘healthcare’ is often implicitly equated with biomedical services, sidelining the diverse sectors of providers that also exist and that people turn to [11]. While biomedical care was frequently perceived as the most accurate and desirable, it was also linked to the highest and most persistent access barriers, echoing other findings that show a disjuncture between institutional prestige and everyday inaccessibility in public health systems [44, 47]. In this context, accessibility and being treated with dignity and respect emerged as conditions for engaging with care. In our study, NGO services appeared to act as a stabilising force and filled critical gaps in care coordination. However, this bridging role also reflects structural deficits in the public system. Reliance on externally funded NGO structures raises concerns about long-term sustainability, equity in service provision and the displacement of responsibility from public institutions [48, 49]. Reforms for equitable care should therefore invest in public system capacity, including referral systems, anti-stigma practices, physical accessibility, affordable transport and transparency around costs, while recognising the plurality of existing care structures. Crucially, this entails understanding how different care providers and systems are evaluated and navigated by users.

### Limitations

The study has several limitations. First, the use of FGDs, while appropriate for capturing shared patterns and collectively negotiated understandings of care-seeking, has limitations: group dynamics and social desirability may have influenced what participants disclosed and, in some cases, individual pathways could not always be traced in full detail. A follow-up study drawing on in-depth interviews is currently underway to address this at the individual level. Second, about half of the participants were recruited through an NGO working in the field of disability care. Although recruitment and data collection were designed to minimise a social desirability bias towards NGO services of ETC (e.g., by conducting data collection through independent local researchers and ensuring data anonymity to participants), this bias cannot be excluded. While speaking or hearing impairments were the only exclusion criteria for the PLWDs, the sample consisted exclusively of people with mobility issues, hence experiences of other PLWDs are not represented in this study. Third, while group composition allowed for comparative insight (e.g., between caregivers with and without long-term NGO support, and between PLWDs and caregivers with CLWDs), a larger study could consider more systematically differences across gender, age, education and income. Fourth, the urban focus of this study, conducted in the capital city of Freetown, limits generalisability: the density and proximity of biomedical and NGO facilities in Freetown is considerably higher than in smaller towns or rural areas. Future research and programme design should therefore pay close attention to structural variation between urban and rural areas. Finally, spiritual care and self-treatment were only marginally represented in the material, limiting a more differentiated analysis of these domains. Future research could explore these domains more systematically, particularly with regard to their role in long-term care trajectories and everyday coping strategies.

## Conclusion

This study highlights how disability-related care trajectories unfold across multiple domains in a pluralistic health system marked by fragmentation and multiple exclusionary mechanisms. Participants’ experiences demonstrate that access to care is a dynamic and situated process shaped by structural and social factors as well as individual agency and abilities. Improving access for PLWDs and their families in a pluralistic system requires investment in public health infrastructure, attention to provider attitudes, affordability, and the social meanings of care. Future research and policy should include local knowledge and lived experiences to design disability-responsive systems that are inclusive, sustainable and aligned with the lived realities of those most affected.

## Supplementary Information

Supplementary Material 1.

## Data Availability

The datasets used during the current study are available from the corresponding author on reasonable request.

## Abbreviations

CLWD: Children living with disabilities
ETC: Enable the children
FGD: Focus group discussion
NGO: Non-governmental organisation
PLWD: People living with disabilities
RQ: Research question
