# Supplementary material for: Disability-related care trajectories in a pluralistic health system: a qualitative study in urban Sierra Leone
Source: BMC Public Health. 2026 Jun 2;26:1781. doi: 10.1186/s12889-026-27861-9 (PMC13231645; doi:10.1186/s12889-026-27861-9)
Supplement: Supplementary file 1 — Supplementary Material 1. [file 12889_2026_27861_MOESM1_ESM.docx]

**Focus Group Discussions with PWDs/Caregivers of PWDs**

1) Healthcare Navigation

- Try to remember – when you first learned that your child/you have a disability, what did you do?
- Whom did you first visit? (probe (traditional/formal) wellbodi people)
- Was there a moment when you felt the care you received was not helping? What did you do then?
- Did you ever seek help from more than one wellbodi place at the same time, or on the same day? Can you tell us about that?
- Were there moments when someone else interfered with or redirected your care-seeking? Who was that? And how did it affect your decisions?
- What steps do you take now when you or the person with a disability in your household need wellbodi business?
- Have you faced any problems in finding the right wellbodi people if you or the person with disability needs care?
- What do you do if you face problems finding the right wellbodi people for yourself or a person with disability? (Probe: What steps do you try next, if any?)
- How are the steps you take now different from what you did at the beginning?
- Are there types of wellbodi business you no longer seek, and why?

2) Long-term Healthcare Seeking

- Are there wellbodi people or places you go to regularly? Why these and not others?
- Have there been times when you stopped looking for wellbodi business altogether? What made you stop?
- How does taking care of this wellbodi business fit into your everyday life over time?
- What makes it easier or harder to continue with wellbodi business for a long time?

3) Barriers and Enablers in Accessing Healthcare

- Can you share your experiences with *X(insert before mentioned wellbodi businesses*) if you or the person with disability needs wellbodi business?
- What are the most important problems you face in accessing wellbodi business?
- How do these problems affect your experience of wellbodi business?
- What changes would make it possible for you to more easily access these wellbodi businesses, or to access other wellbodi businesses that you feel you may need?

4) Current status of healthcare for PWDs

- Have wellbodi businesses for PWDs improved in Sierra Leone in recent years? If yes, what has improved? What still needs to change?
- How can wellbodi businesses better address your needs?
- Looking back, if someone else in your situation asked you where to start and what to avoid, what would you tell them?

Corresponding table to Figure 2

|  | **At symptom onset** | | | **Long-term care** |
| --- | --- | --- | --- | --- |
|  | First step | Second step | Third step |  |
| Biomedical care | 32 | 15 | 13 | 12 |
| Traditional care | 8 | 8 | 1 | 0 |
| Spiritual care | 5 | 4 | 0 | 5 |
| Social network support | 3 | 0 | 0 | 2 |
| NGO clinic/ services | 2 | 4 | 5 | 14 |
| No care seeking | 2 | 0 | 0 | 3 |
